# PTEN as a Prognostic/Predictive Biomarker in Cancer: An Unfulfilled Promise?

**DOI:** 10.3390/cancers11040435

**Published:** 2019-03-28

**Authors:** Chiara Bazzichetto, Fabiana Conciatori, Matteo Pallocca, Italia Falcone, Maurizio Fanciulli, Francesco Cognetti, Michele Milella, Ludovica Ciuffreda

**Affiliations:** 1 Medical Oncology 1, IRCCS - Regina Elena National Cancer Institute, Rome 00144, Italy; chiara.bazzichetto@ifo.gov.it (C.B.); fabiana.conciatori@ifo.gov.it (F.C.); italia.falcone@ifo.gov.it (I.F.); francesco.cognetti@ifo.gov.it (F.C.); 2 Department of Molecular Medicine, University of Rome, La Sapienza, Rome 00185, Italy; 3 SAFU, Department of Research, Advanced Diagnostics, and Technological Innovation, IRCCS - Regina Elena National Cancer Institute, Rome 00144, Italy; matteo.pallocca@ifo.gov.it (M.P.); maurizio.fanciulli@ifo.gov.it (M.F.); 4 Section of Oncology, Department of Medicine, University of Verona School of Medicine and Verona University Hospital Trust, Verona 37126, Italy; michele.milella@univr.it

**Keywords:** PTEN, biomarkers, personalized cancer therapy

## Abstract

Identifying putative biomarkers of clinical outcomes in cancer is crucial for successful enrichment, and for the selection of patients who are the most likely to benefit from a specific therapeutic approach. Indeed, current research in personalized cancer therapy focuses on the possibility of identifying biomarkers that predict prognosis, sensitivity or resistance to therapies. Phosphatase and tensin homolog deleted on chromosome 10 (PTEN) is a tumor suppressor gene that regulates several crucial cell functions such as proliferation, survival, genomic stability and cell motility through both enzymatic and non-enzymatic activities and phosphatidylinositol 3-kinase (PI3K)-dependent and -independent mechanisms. Despite its undisputed role as a tumor suppressor, assessment of PTEN status in sporadic human tumors has yet to provide clinically robust prognostic, predictive or therapeutic information. This is possibly due to the exceptionally complex regulation of PTEN function, which involves genetic, transcriptional, post-transcriptional and post-translational events. This review shows a brief summary of the regulation and function of PTEN and discusses its controversial aspects as a prognostic/predictive biomarker.

## 1. Introduction

Cancer is a very complex and dynamic disease, characterized by both genetic and microenvironmental features. Decades of genetic studies have allowed the identification of a landscape of somatic mutations in a large collection of genes involved in tumor development. Recently, Sondka et al. described 719 cancer-associated genes in humans: 574 genes were classified in the catalogue of somatic mutations in cancer Tier 1 genes (genes whose mutation is associated with the promotion of neoplastic transformation) and 145 in Tier 2 genes (genes that show a role in the development of neoplasia, but their role is not completely identified) [1]. Moreover, it has become clear that malignant tumors are highly heterogeneous, not only in different patients with the same histologic tumor type (interpatient heterogeneity), but also in the same single tumor mass or in different metastatic lesions from the same primary tumor in a single patient (intrapatient heterogeneity). Thus, therapeutic targets are not uniformly present throughout the tumor cell population [2,3].

The identification of new biomarkers and the development of gene expression signatures are necessary to improve early diagnoses and accurate prognoses, as well as to provide an opportunity to better match the most effective drugs with the molecular characteristics of each individual patient. Studies over the years have clearly indicated that prognostic and predictive biomarkers are often molecules involved in the regulation of cellular mechanisms, including proliferation, apoptosis, angiogenesis, metastasis and in therapeutic resistance [4]. Accumulating evidence has shown that PTEN is an important factor that regulates many of the processes related to cancer development and progression [5]. 

Germline mutations of the PTEN gene cause the well-known PTEN hamartoma tumor syndromes. These syndromes, which are molecularly characterized, include Cowden, Proteus, Proteus-like and Bannayan–Riley–Ruvalcaba syndromes [6]. PTEN germline mutations are also found in neurological and developmental disorders such as autism spectrum disorder [7,8]. Moreover, PTEN mutations are frequently observed in a wide variety of sporadic cancers, such as glioblastoma (GBM) (20%), malignant melanoma (15%), endometrial (20%), prostate (20%), breast (7%), colorectal (6%) and pancreatic (1.4%) (data obtained from genomic datasets in the cBioPortal for Cancer Genomics are reported in Table 1) [9,10].

Moreover, loss of PTEN tumor suppressor activity may represent a cancer vulnerability, which could be exploited therapeutically. Indeed, our group has recently shown that combined mitogen-activated protein kinase (MAPK) and PI3K inhibition is selectively synergistic in tumors with PTEN loss. Therefore, the recent failure of mitogen-activated protein kinase kinase (MEK)-/AKT-inhibition combination in advanced pancreatic cancer could have been predicted preclinically. All this evidence suggests an urgent need to devise more efficient translational strategies to bring new therapies to the patients’ bedsides in pancreatic and other cancers [11,12,13].

Despite its undisputed role as a tumor suppressor, assessment of PTEN status in sporadic human tumors has yet to provide clinically robust prognostic, predictive or therapeutic information. This is possibly due to the exceptionally complex regulation of PTEN function, which includes, for example, transcriptional, post-transcriptional and post-translation mechanisms. Thus, loss of PTEN function bears a great, but yet unfulfilled, potential as a prognostic and predictive biomarker to prospectively select groups of patients at the highest chance of benefit for specific therapeutic interventions. In this review, we summarize recent advances in PTEN regulation and function, specifically focusing on relationships with cancer clinicopathological features and on factors that could make PTEN a new biomarker for diagnosis, prognosis and prediction of response in cancer therapy.

## 2. Multiple Layers of PTEN Regulation

**Figure 1:**
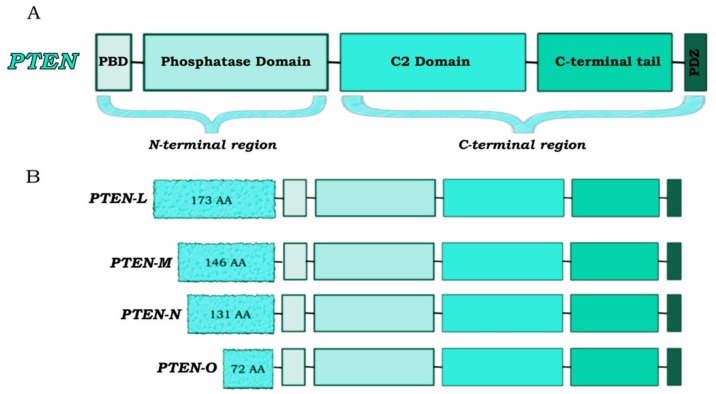
PTEN protein structure. (**A**) The canonical PTEN isoform consists of 403 amino acids structured in five functional domains: a phosphatidylInositol-4,5-bisphosphate (PIP2)-binding domain (PBD), a phosphatase domain (catalytic core), a C2 lipid membrane-binding domain, a C-terminal tail domain (which contains phosphorylation sites implicated in the regulation of PTEN activity/stability and localization) and a PDZ protein–protein interactions domain. (**B**) PTEN has four translational variants (PTEN-L, -M, -N and -O) which contain an additional N-terminus and differ in functions and sub-cellular localization. Translation begins from a translation site upstream the canonical initiation sequence and resulting in N-terminal extensions for each of the variants.

PTEN is a tumor suppressor gene mapping to chromosomal region 10q23 and encoding for a 403-amino acid protein that plays a pivotal role in signal transduction (Figure 1) [14]. 

Loss of PTEN function can occur through a mix of genetic/epigenetic mechanisms including point mutations, chromosomal deletions, promoter hypermethylation and post-translational modifications, leading to a wide spectrum of human diseases [15]. Furthermore, PTEN is a haploinsufficient tumor suppressor. Indeed, partial loss of PTEN function is sufficient to promote tumor development, and a 50% reduction in PTEN levels is associated with further acceleration of cancer progression [16].

The PTEN gene contains 9 exons, which harbor many variations at the germline level. Even if a lot of those are found in exon 5 (which encodes for the phosphatase core motif) and the other relevant variations are clustered mainly in exon 7 and 8, they are scattered along the whole gene [17]. PTEN mutations comprise point missense mutations, stop-gain mutations, small deletions, insertions and splice site mutations (Table 1) [9,10].

Transcriptional regulation of PTEN occurs through both epigenetic and transcriptional mechanisms. Epigenetic modulation of PTEN expression leads to the silencing of the gene through the presence of hypermethylated CpG islands. This mechanism of silencing is observed in numerous cancers (e.g., leukemia, colorectal cancer (CRC), hepatocellular carcinoma, melanoma, breast and thyroid cancers) [18,19]. PTEN is also negatively and positively regulated by different transcription factors. Negative regulators can directly (such as c-JUN by binding to a variant AP-1) and indirectly (such as nuclear factor-kappa B activated by mitogen-activated protein kinase kinase-4) inactivate PTEN expression; positive regulators include different proteins such as TP53 and early growth regulated transcription factor 1 [20]. 

MicroRNAs (miRNAs) are critical regulators of PTEN abundance at the post-transcriptional level. Different miRNAs can increase or decrease PTEN levels—including those derived from miRNA precursors containing a single hairpin structure (e.g., miR-21, miR-22 and miR-205), as well as those with a polycistronic structure (e.g., mir-17-92, mir-106b-25, mir-367-302b and mir-221-222)—thus playing a role as a tumor suppressor or tumor promoter, respectively [21]. For example, miR-25 overexpression, caused by MAPK’s activation, negatively regulates PTEN protein expression in human tumors [22]. PTEN post-transcriptional regulation also occurs through the activity of different isoforms of the sense and antisense as long non-coding RNA PTENpg1. Indeed, PTENpg1 asRNA *α* and the unspliced nuclear localized PTENpg1 asRNA *α* suppress PTEN transcription, whereas sense PTENpg1 promotes PTEN transcription. Sense PTENpg1 binds PTENpg1 asRNA *β*, thereby increasing the activity of sense PTENpg1. Sense PTENpg1 binds miRNA targeting of PTEN with its 3′ UTR, thereby increasing PTEN messenger RNA stability and the consequent amounts of the protein [23].

In addition, numerous post-translational modifications (such as ubiquitination, acetylation, sumoylation and phosphorylation) modify the properties of PTEN as a tumor suppressor and impact PTEN localization in the cell [24,25,26]. Monoubiquitination or ubiquitination of specific residues (such as Lys13 and Lys289) are involved in PTEN shuttling and nuclear import, while PTEN polyubiquitination leads to PTEN cytoplasmic retention and degradation [27]. Although the acetylation of Lys402 does not cause a change in the intracellular localization of PTEN, this modification enhances the interaction of PTEN with PDZ-domain proteins, leading to stronger interactions with the plasma membrane [28]. While sumoylation is essential to PTEN tumor suppressive functions, phosphorylation in the C-terminal cluster of Ser and Thr residues maintains PTEN in a closed conformation by increasing the anchorage to the cell membrane, reducing phosphatase activity and membrane localization [29,30,31]. The deletion of PTEN C-terminal tail region results in a lower stability of the protein due to the increasing of its degradation, but not in a lower activity as tumor suppressor, thereby reflecting the inhibitory role of the tail in PTEN regulation [32]. Moreover, Chen et al. have recently demonstrated, through protein mutagenesis, photo-cross-linking and semisynthesis experiments, that although at least three phosphorylation sites (Ser380, Thr382, Thr383 and Ser385) are needed to mimic PTEN lipid phosphatase inactivation, each phosphorylation site can contribute to increasing the stability of closed conformation PTEN [33]. PTEN C-terminal tail is phosphorylated by different kinases: casein kinase 2, glycogen synthase kinase 3 β, FYN-related kinase (also known as RAK), Protein Interacting with Carboxyl Terminus (PICT)-1 and rho-associated kinase [18,34].

**Figure 2:**
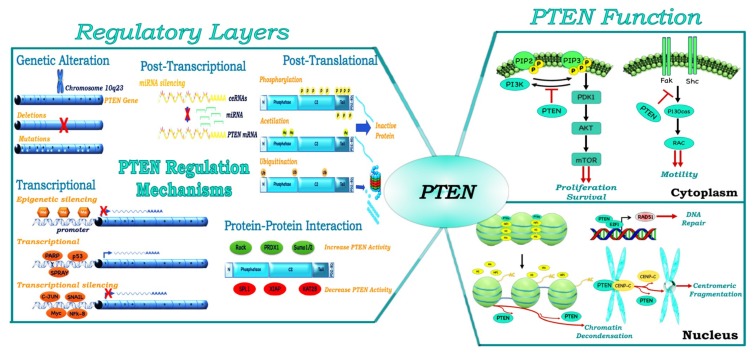
PTEN protein regulation and function. PTEN protein expression is regulated by genomic (mutation and deletions), transcriptional (epigenetic mechanisms and transcription factors), post-transcriptional (miRNAs, PTEN pseudogene), post-translational mechanisms (phosphorylation, acetylation, ubiquitination, etc.) and protein–protein interaction. PTEN performs multiple cellular functions, at least in part determined by its subcellular localization. In the cytoplasm, PTEN dephosphorylates PhosphatidylInositol-3,4,5-trisPhosphate (PIP3), decreasing PI3K activity and several proteins such as focal adhesion kinase (FAK) or Src-Homology/Collagen (SHC), to regulate different signal networks. The nuclear PTEN activities include the regulation of genomic stability, gene expression, DNA repair mechanisms and centromeric stability.

Increasing evidence also shows that PTEN can interact with both itself and other protein partners, modulating its activity [35]. Pandolfi et al. demonstrated that PTEN homodimerization enhances its lipid phosphatase function through C-terminal tail stabilization [36]. Whereas PTEN homodimerization seems to be a positive mechanism of activation, the interaction between PTEN and other proteins can modulate PTEN activity and subcellular localization both positively and negatively [37]. For example, PICT1 (also known as NOP53) and RAK physically bind to PTEN and promote its phosphorylation and stabilization. Conversely, the binding of proteins like Shank-Interacting Protein 1 (also known as SHARPIN) and α-mannosidase 2C1 suppresses PTEN lipid phosphatase activity [38,39,40]. The fine mechanisms of PTEN gene/protein regulation are summarized in Figure 2.

## 3. PTEN, a Tumor Suppressor and More

PTEN acts as a non-redundant negative regulator of the PI3K/AKT pathway with a specificity for both protein and lipid substrates. The two main functional domains identifiable in the PTEN structure are the phosphatase domain and the C2 domain. While the phosphatase domain is responsible for enzymatic activity of the tumor suppressor, the C2 domain allows its membrane association by promoting the positioning of the catalytic domain on the plasma membrane [41]. C-terminal tail, which contains a PDZ-binding protein domain, is implicated in the regulation of PTEN activity/stability. Indeed, proteins that interact with PTEN through the PDZ domain are involved in PTEN stabilization and in its specific subcellular compartmentalization (Figure 1) [42,43]. The main function of PTEN is its lipid phosphatase activity: Class I PI3Ks catalyze PIP2 phosphorylation to generate the lipid signaling intermediate PIP3, and PTEN antagonizes PI3K activity by dephosphorylating PIP3 [44]. Moreover, the lipid phosphatase activity of PTEN has been demonstrated to be critical for its tumor suppressing function [45]. Through its protein phosphatase activity, PTEN is able to dephosphorylate itself and other protein substrates, including membrane-bound, cytoplasmic and nuclear proteins, thus regulating several biological functions including cell cycle progression and migration [46]. For example, Tamura et al. demonstrated that focal adhesion kinase, which is directly involved in cellular adhesion, is dephosphorylated by PTEN, thereby inhibiting cell migration and invasion [47]. 

PTEN also has several lipid- and phosphatase-independent functions involved in cell-cycle regulation, migration and tumor progression [24,48]. Indeed, according to its subcellular compartmentalization (e.g., nucleus, mitochondria and endoplasmic reticulum), PTEN displays additional functions. For example, nuclear PTEN plays a central role in maintaining the stability of genetic information by interacting with key molecules involved in DNA replication, as well as controlling chromosome segregation during cell cycle progression. Indeed, it has been demonstrated that PTEN is involved in centrosome stability through binding to the centromeric protein C (CENP-C). Moreover, PTEN regulates Rad51 transcription, one of the principal components of the DNA repair family with BRCA1 and BRCA2 (Figure 2) [49]. However, the activation of Rad51 requires the direct association between PTEN and the E2F-1 transcription factor to its promoter, thereby suggesting the importance of synergistic interaction between the two elements in mediating this process [49]. Moreover, through the interaction with p53, PTEN cooperates with the tumor suppressor during oxidative stress, thereby causing cell-cycle arrest [50]. Recent evidence has also revealed an association between nuclear PTEN and splicing machinery, thus affecting the regulation of the alternative splicing of numerous pre-mRNAs [51].

In addition, distinct functions of PTEN are associated with the extracellular environment through its secretion and export. Cellular non-autonomous effects of PTEN are mediated by two main mechanisms, direct (through the release from the cell of a membrane-permeable PTEN isoform (PTEN-L)) and indirect (through the release of PTEN into exosomes) [37]. PTEN-L is the most studied proteoform and is generated from a translational variant of PTEN mRNA. In addition, three other variants have been described (PTEN-M, PTEN-N and PTEN-O), but their role is yet to be elucidated [52]. The presence of a membrane-binding helix in these proteins could lead to their association with the membrane. Moreover, PTEN-L and PTEN-M could play a role in the nucleus and nucleolus due to a putative sequence of nuclear localization [53] (see Figure 1).

Recent evidence has also highlighted a potential role of PTEN in regulating tumor microenvironment (TME) features. Experiments in mice demonstrated that deletion of PTEN in fibroblasts creates a tumor-permissive stroma in mammary gland tumors, with a consequent recruitment of innate immunity cells, remodeling of the extracellular matrix and tumor vasculature, thus increasing malignancy [54]. TME remodeling is also influenced by PTEN activity through the modulation of chemokine/cytokine production. For example, a correlation between PTEN loss and interleukin (IL)-8 upregulation in prostate carcinoma and GBM has been described [55,56]. Moreover, PTEN-loss senescent cells secrete immunosuppressive chemokines through the activation of janus-activated kinase 2/signal transducer and activator of transcription 3, leading to an immunosuppressive TME in prostate cancer [57]. Numerous studies revealed the active contribution of PTEN to the regulation of angiogenesis at different levels. PTEN-loss cancer cells display increasing levels of the expression of vascular endothelial growth factor due to the upregulation of hypoxia-inducible factor 1 [58]. In addition to its role in angiogenesis, PTEN may also regulate cell migration through modulation of the expression of matrix metalloproteinase (MMP). Indeed, PTEN overexpression in multiple myeloma cell lines resulted in decreased mRNA and protein expression of MMP-2, MMP-9 and Focal Adhesion Kinase (FAK), thus leading to decreased cell migration ability [59].

Several studies have shown that the PI3K/AKT pathway can modulate immune cell activities, thereby impacting the potential effectiveness of cancer immunotherapy [60]. Among the most studied molecules, programmed death-1 (PD-1), and its ligand, PD ligand-1 (PD-L1), have been clinically validated as relevant therapeutic targets. It has been demonstrated that loss of the tumor suppressor PTEN can induce PD-L1 overexpression in several cancers, such as pancreatic, colorectal and breast cancer [61,62,63,64,65]. This relationship highlighted the ability of PTEN to affect resistance not only to conventional chemotherapy and/or targeted therapies but also to immunotherapy [66,67].

## 4. PTEN as a Prognostic Biomarker

Over the last decades, a wealth of prognostic and/or predictive biomarkers have been studied with the aim to more accurately predict outcomes of individual patients, on the one hand, and to improve treatment response on the other. A handful of such predictive biomarkers have proven so crucial in the cancer drug development process that specific tests to accurately measure relevant biomarkers have been approved by regulatory agencies together with their matching drugs, so that selected drugs can be prescribed only in biomarker-positive patients (companion diagnostics) [68,69,70,71,72]. As mentioned above, despite its undisputed relevance to cancer biology and potentially to clinical tumor behavior, the role of PTEN as a prognostic and/or predictive biomarker remains controversial. Indeed, currently no specific, validated test is available to assess PTEN protein functionality/activity. Due to the complex regulation described above, the mere mutational analysis of the gene or expression of it is not sufficient to predict a lack of PTEN function (PTEN loss status). Such difficulties in assessing the functional status of PTEN could in turn explain discrepancies observed in regard to PTEN prognostic/predictive value in different studies of the same types of cancer. 

### 4.1. Urogenital Cancers

#### 4.1.1. Prostate

PTEN loss has been found to occur in approximately 20% of prostate tumors, and its mutation or deletion is currently recognized as one of the critical factors in hormone-naïve and castration-resistant prostate cancer [73]. Recent studies have highlighted the association between PTEN loss and not only a higher grade of aggressiveness (measured by the Gleason score) and higher probability of invasion and metastasis, but also an increased risk of death [74,75]. In addition, a recent study showed that patients affected by prostate cancer and treated with radical prostatectomy displayed a significant correlation with a higher risk of recurrence in the absence of PTEN protein (assessed by immunohistochemistry (IHC) analysis) expression [76]. 

Given its role in genomic stability, PTEN loss also correlates with higher levels of chromosome instability and increased aneuploidy in prostate cancer. Viadotto et al. recently demonstrated that the mechanisms of PTEN deletion are not random, but fall into five distinct classes with a conserved clustering of somatic copy number alterations and specific acquired features [77]. Preclinical data have also suggested that large PTEN interstitial deletions are characterized by higher levels of TP53 mutations as compared to other classes of PTEN defects, and are associated with increased angiogenesis, migration and tumor cell stemness—all traits that confer more aggressiveness to this tumor subtype [77].

#### 4.1.2. Kidney

Que et al. have recently carried out a meta-analysis identifying PTEN loss as a prognostic factor for worse overall survival (OS) in patients affected with kidney cancer due its role in increasing aggressiveness and the progression of this type of tumor [78]. Tang et al. have also shown a statistically significant association between low levels of PTEN and unfavorable disease-specific survival in patients with Renal Cell Carcinoma (RCC), a specific subtype of kidney cancer [79]. However, in this series PTEN expression did not correlate with other clinicopathological features such as Fuhrman grade, pathological type (Clear Cell (CC)RCC vs. other RCC types) or clinical stage, thus rendering PTEN a good prognostic, but not predictive, biomarker for kidney cancer [79]. Consistent with this observation, Abou Youssif demonstrated that there is no association between metastatic RCC lesions and mutations in the PTEN gene [80]. Moreover, biallelic loss of PTEN was shown to be associated with worse OS, as compared to its monoallelic defect [35]. However, in a different series the prevalence of PTEN mutations in primary RCC tumors was extremely variable (ranging from 4% to 42%), thus making it difficult to attribute a prognostic role to PTEN gene status. Moreover, during cancer development clonal events characterize genetic tumor heterogeneity, thus rendering it cumbersome to identify common features that can be used as good biomarker(s). Indeed, a study on 101 patients with CCRCC investigated subtypes of patients with different clinical phenotypes of the disease. Among these, a ‘‘multiple clonal driver” stage III+ subtype harbored PTEN mutations and displayed an increase in Ki67 percentage, thus suggesting PTEN status as a potential biomarker in a subset of CCRCC patients [81].

### 4.2. Gastrointestinal Cancers

#### 4.2.1. Pancreas

Several data have shown that PTEN mutations are associated with pancreatic cancer development in animal models and that, even if its mutation is rarely identified in pancreatic cancer, PTEN protein expression is often low or completely absent [82,83,84]. Moreover, immunohistochemical analysis carried out on patient samples showed that higher PTEN expression was evident only in patients without liver metastases (low Tumor, Node, Metastasis (TNM) stage), as compared to those with metastases [85]. Foo et al. also demonstrated that patients whose tumors displayed PTEN loss had a higher proportion of recurrence/metastases. These patients had a shorter OS, as compared to PTEN-competent ones, but no association with other clinicopathological parameters were observed [86]. Conversely, Pham previously reported that higher levels of PTEN expression were associated with pancreatic ductal adenocarcinoma (PDAC) progression in 26 cases of Tissue MicroArray (TMA)s, thus suggesting that the number of cases could interfere with the outcome of PTEN analysis [87].

#### 4.2.2. Colorectal

CRC is one of the main causes of cancer death and is often associated with the mutation of oncogenes and tumor suppressors downstream the epidermal growth factor receptor (EGFR) such as BRAF, Kirsten rat sarcoma (KRAS), PI3KCA and PTEN [88]. CRCs can be classified through four main “features”: microsatellite instability (MSI) and chromosomal instability, as well as global genomic status and CpG island methylator phenotype (CIMP) status for epigenomic status [89]. The loss of PTEN expression does not seem to be related to MSI and CIMP status, thus discouraging the use of PTEN loss as a prognostic factor in CRC diagnosis [90]. However, the role of PTEN as a prognostic biomarker in CRC remains controversial. Indeed, several studies have shown that loss of PTEN expression is associated with liver and lymph node metastases, and correlates with a significant reduction in patients’ survival; however, the loss of nuclear PTEN is also a marker of poor clinical outcome [91,92]. Moreover, Jang demonstrated that nuclear PTEN expression is observed only in patients with normal colorectal epithelium, and is not detectable during the neoplastic transformation processes [92]. Yazdani et al. also demonstrated that, during CRC growth and invasion and metastatic events, the PTEN promoter is methylated and miR21 levels increase, thus leading to the down-regulation of PTEN expression [93]. All these provide evidence that regulation of PTEN expression could be a good target for pharmacological intervention in CRC treatment.

### 4.3. Breast Cancer

Somatic PTEN mutations are rare (around 5%) in sporadic breast carcinoma; however, the frequency of PTEN gene loss is approximately 30%–40%, and accounts for approximately 20%–25% of HER2-positive breast cancers [94,95]. Indeed, even if PTEN mutations are rare in breast cancer patients, protein expression is absent in up to 48% of cases [96]. Moreover, it has been demonstrated that detection of PTEN through mRNA levels is a good predictor of PI3K pathway activation, as compared to protein IHC analysis [96]. The activation of the PI3K pathway, due to PTEN mutation or absence correlates with tumor progression and poor prognosis in breast cancer [94,96]. Recent studies by Golmohammadi et al. have shown a significant association between the invasiveness of ductal tumors and the lack of the PTEN gene as compared to ductal tumors [97]. Specifically, complete loss of PTEN in sporadic invasive ductal carcinomas is significantly associated with estrogen receptor negativity [98].

### 4.4. Endometrial Cancer 

EC is one of the most common female neoplasia. PTEN mutations are found in approximately 25% of cases of hyperplasia and up to 80% of endometrioid EC (EEC) cases [99,100]. In particular, EEC is one of the most common types of endothelial carcinoma, and is characterized by microsatellite instability and mutation in KRAS, PI3KCA and PTEN [101]. The expression level of PTEN mRNA and protein appears to be significantly lower in endometrial cancer tissue as compared to normal endometrial tissue. This could in turn make PTEN loss a biomarker for early diagnosis during the development of endometrial cancer [102]. According to these findings, the European Society of Gynaecological Oncology recommends the use of PTEN IHC as a valid criterion for the early differential diagnosis between benign and premalignant hyperplasia. Alternatively, Raffone et al. conducted the first meta-analysis to investigate the usefulness of PTEN IHC as a good prognostic biomarker, and suggested that PTEN IHC should be reconsidered, highlighting the necessity to improve its diagnostic accuracy [103].

### 4.5. Brain Cancers

Glioma represents the most frequent (around 40%) brain neoplasm of the central nervous system, displaying a very high incidence of PTEN gene mutations (from 25% to 40% of glioma cases). Given the key role of PTEN activity in physiological mechanisms of the development of brain cells and of the maintenance of cerebral homeostasis, its activity has also been investigated in processes such as tumor progression and grade of glioma malignancy [104,105]. Consistent with PTEN functions in brain homeostasis, Yang and collaborators showed that PTEN mutations often occur at the phosphatase and C2 domains, thereby leading to the hyperactivation of the PI3K signaling pathway [106]. Furthermore, it has been demonstrated that PTEN mutation is a late event in glioma progression, highlighting the inverse correlation of PTEN expression with WHO grade tumors [107]. According to this body of evidence, recent meta-analysis studies have indicated that PTEN mutation is associated with poor prognosis and shorter survival time in glioma patients [84,108]. 

### 4.6. Skin Cancers

PTEN gene alteration is not among the most frequent mutational events in melanoma (10%), but PTEN loss co-occurs with BRAF activating mutations (the key driver abnormality in melanoma) in 44% of BRAF mutant melanoma [109]. Despite the role of PTEN as a haploinsufficient tumor suppressor, only complete absence of the protein is associated with a reduced OS in BRAF-mutant patients [110]. The clinical evaluation of PTEN protein in patients is often carried out by IHC; however, IHC is not the only detection method to identify a correlation between PTEN loss status and patient prognosis. Indeed, Giles et al. demonstrated that an analysis of PTEN epigenetic modifications could also identify patients with an inactive PTEN and a worse prognosis [111]. More specifically, methylation of the PTEN gene, which is associated with gene silencing, is significantly correlated with excess deaths when analyzed in the context of specific prognostic characteristics (e.g., mitotic index, tumor surface diameter and age) [112].

### 4.7. Hematological Cancers

The PI3K/mammalian target of rapamycin (mTOR) pathway is one of the most frequently deregulated pathways in leukemia. Even though PTEN mutations are not frequently observed in myeloid malignancies (1%), PTEN protein is often inactivated in acute myeloid leukemia (AML) and chronic myeloid leukemia [113]. Cheong et al. showed that patients with phospho-PTEN have a shorter OS as compared to non-phospho-PTEN patients, demonstrating that PTEN inactivation, mediated by its C-tail phosphorylation, could be a poor prognostic factor in AML [114].

The inactivation of the PTEN gene also occurs in many cases of pediatric and adult T cell acute lymphoblastic leukemia. Paganin et al. recently demonstrated that PTEN mutations, alone or in combination with NOTCH1 mutations, might identify patients with a higher probability of relapsing [115]. Nevertheless, different studies indicate that PTEN was not a strong biomarker. Indeed, PTEN mutation is often not sufficient per se to predict the evolution of a particular patient cohort [116]. 

### 4.8. Pan-Cancer Overview

In order to present the current status of PTEN loss as a prognostic biomarker in currently available genomic and clinical data, we performed an automated survival analysis on all 31 datasets of The Cancer Genome Atlas (TCGA) through the cBioPortal R package cancer genomics data server at the time of the writing (Table 2) [9,10,117]. Duplicated dataset entries were excluded by keeping only those studies with the highest number of patients. The endpoint of the survival analysis was overall survival (OS) and disease-free survival (DFS) (when available), and all datasets were partitioned by deep PTEN deletions (i.e., PTEN loss, Genomic Identification of Significant Targets In Cancer (GISTIC) score < −1) or with deep and shallow PTEN deletions (i.e., PTEN Del, GISTIC score < 0) [118]. In the control group, all patients harboring PTEN mutations were excluded to reduce confounding factors of loss or gain or function events. Results showed that, in a subset of cancer types, the effect of PTEN deletions is strongly associated with a shorter OS/DFS. In three particular scenarios—lower grade gliomas (LGG), uterine corpus EC (UCEC) and glioblastoma (GBM)—the effect was significant on OS and DFS for both PTEN loss and deletions.

Overall, among 124 tests of survival separations for 31 datasets and 4 conditions (PTEN loss/del and OS/DFS), 13 did not have available clinical data and only a subset of 26 combinations resulted significantly in separating survival curves (*p* < 0.05). 

We focused on genomic deletions in order to capture the most disruptive events occurring at the genomic level, excluding point mutations of uncertain significance. 

Finally, we reported the most significant analyses of survival median values in terms of OS and DFS in Table 3.

## 5. PTEN as a Predictive Biomarker

In addition to its role as a potential prognostic biomarker, the loss of PTEN could be also used as a predictor for drug response in several tumors including prostate, breast, endometrium and many others. Indeed, a good predictive biomarker aims to objectively identify the patients who are most likely to benefit from specific therapeutic approaches [119].

Notable from the relatively high percentage of PTEN loss in prostate cancer, several groups have investigated its potential role in the response to radiotherapy, targeted therapies and conventional chemotherapy, trying to overcome the absence of a uniformly accepted assay to measure PTEN levels [73]. Recently, de Bono et al. demonstrated that PTEN-loss patients better respond to combined therapy consisting of the ATP-competitive AKT inhibitor ipatasertib with abiraterone, as compared to PTEN-competent patients (identified by IHC) [120]. Punnoose et al. previously showed that PTEN status could be a predictive biomarker in combination trials of abiraterone and PI3K/AKT inhibitors, with also another PTEN detection technique. However, in contrast with de Bono’s data, Punnoose demonstrated that PTEN loss, analyzed by both blood-based PTEN fluorescence in situ hybridation (FISH) assay in circulating tumor cells and IHC, correlates with a poorer clinal outcome in castration-resistant prostate cancer, possibly due, at least in part, to the small sample size used [121]. Fontugne et al. also analyzed the correlation between PTEN status and radiotherapy outcome. They demonstrated that patients with PTEN deletion, identified by both IHC and FISH analysis, displayed significantly worse relapse-free survival rates compared to those with wild-type PTEN [122].

PTEN loss status is a good prognostic biomarker of sensitivity to mTOR complex 1 inhibitor everolimus in vitro; however, several clinical trials failed to demonstrate this correlation [123,124]. Indeed, TAMRAD and BOLERO-2 trials showed that PTEN loss does not correlate with the clinical outcome of everolimus treatment in breast cancer patients [125,126].

This controversial role of PTEN as a positive or negative prognostic biomarker has also been observed in HER2-positive breast cancer patients treated with chemotherapeutic agents plus anti-HER2 therapy (trastuzumab). Indeed, even though trastuzumab is highly effective in patients, many HER2- positive tumors become resistant to treatment and, therefore, it is necessary to identify other biomarkers [95]. Different groups demonstrated that PTEN loss status is a predictor for trastuzumab resistance, otherwise low PTEN levels are associated with a response to lapatinib [127,128]. Indeed, Fabi et al. demonstrated that the co-expression of PTEN and phospho-AKT can identify a subset of HER2-positive patients with an increased chance of benefitting from trastuzumab therapies in metastatic breast cancer [129].

Monoclonal antibodies for EGFRs are also clinically used for metastatic CRC (mCRC), and several groups demonstrated that PTEN expression also predicts clinical outcome in this histotype. Indeed, Frattini’s group showed that PTEN-loss mCRC patients do not benefit from cetuximab treatment. Two years later, Loupakis et al. demonstrated that PTEN loss in metastases could also be predictive for the combined treatment outcome of cetuximab plus irinotecan [130,131]. These data suggest that among the wild-type KRAS tumors, it could be useful to identify PTEN status in order to choose the subtype of patients who can benefit the most from specific treatments.

PTEN status could be a predictor not only for targeted therapies plus chemotherapy, but also of different targeted therapy combination outcomes. Chung et al. recently showed that the dual targeting of MEK and PI3K/AKT signaling does not exhibit clinical benefits in unselected PDAC patients [11]. We demonstrated that the combination of MEK and PI3K inhibitors is effective only in PTEN loss contexts, thus explaining the failure of the combination of selumetinib and MK-2206 in unselected PDAC, leading to a dire need to identify new biomarkers [13]. Indeed, our group has recently demonstrated that PTEN plays an important role in determining the functional outcome of combined MAPK/PI3K inhibition in vitro. Combined treatment results in synergistic effects on tumor growth only in PTEN-loss cell lines with different histological origins. This result was further confirmed using the isogenic CRC cell line HCT116 with homozygous knock-out of the PTEN gene. The isobologram analysis revealed additive/antagonistic interactions in parental HCT116 cells and strong synergistic effects in PTEN knock-out isogenic cell lines [12]. Moreover, PTEN status could influence the response to targeted therapies in the presence of stromal and TME elements. The preliminary results obtained by our group showed that TME features do not affect the response to MEK inhibitors, whereas the response to PI3K inhibition is strongly influenced by TME. Indeed, soluble factors released by fibroblast cells profoundly increase sensitivity to PI3K inhibitors only in PTEN-competent cell lines, as compared to PTEN-loss cell lines [132]. 

## 6. Conclusions

PTEN is a haploinsufficient tumor suppressor that regulates several cell functions (i.e., proliferation, survival, genomic stability and cell motility) and it is mutated in several types of cancer. PTEN displays a very complex mode of regulation that makes identifying clinical situations in which loss of PTEN activity is a major driving force particularly cumbersome. To date, there is no clinically validated detection assay to analyze PTEN expression and function, but an increasing number of studies have highlighted its potential role as a prognostic and predictive biomarker in cancer. The development of a functional “signature” of loss of PTEN activity would be the most innovative approach, in order to overcome the limitation of functional PTEN detection and to identify genes, miRNAs or proteins that could directly recognize defective PTEN function.

## Figures and Tables

**Table 1:** PTEN mutations in different tumor histotypes.

Site	Tumor Type	Range	Average	n Sample	n TCGA Studies	Comments
Kidney	CCRCC	3–5%	4%	1548 patients/1549 samples	3	Nonsense, missense, FS ins, FS del, splice, IF del
	Non-CCRCC	2.8–11%	5%	772 patients/773 samples	5	Nonsense, missense, FS del, IF del
Prostate	Adenocarcinoma	17–21%	20%	1325 patients/1326 samples	3	Nonsense, missense, FS ins, FS del, splice, IF del, fusion
Pancreas	Adenocarcinoma	1.1–1.6%	1.4%	369 patients/370 samples	2	NA
Colorectal	Adenocarcinoma	4–8%	6%	1506 patients/1510 samples	3	Nonsense, missense, FS ins, FS del, splice
Breast	Invasive Carcinoma	4–11%	7%	3824patients/3832 samples	4	Nonsense, missense, FS ins, FS del, splice, IF del
Ovary	Serous Cystadenocarcinoma	6–7%	7%	1742 patients/1754 samples	3	Missense, FS del, splice
Uterus	EC	21%	21%	114	2	Nonsense, missense, FS ins, FS del, splice
CNS–Brain	Diffuse glioma	4–15%	10%	2152 patients/2168 samples	3	Nonsense, missense, FS ins, splice, nonstart, fusion
	GBM	19–32%	22%	1967 patients/1987 samples	4	Nonsense, missense, FS ins, FS del, splice, IF del
Skin	Melanoma	14–16%	15%	913 patients/927 samples	2	Nonsense, missense, FS ins, FS del, splice, IF del, fusion
Myeloid	AML	1%	1%	200 patients/200 samples	3	NA
Thyroid	Carcinoma	1.2%	1.2%	1514 patients/1523 samples	3	Nonsense, missense, FS del, fusion

PTEN, Phosphatase and tensin homolog deleted on chromosome 10; TCGA, the cancer genome atlas; NA, not available; FS ins, frameshift insertion; FS del, frameshift deletion; IF del, in-frame deletion; CCRCC, clear cell renal cell carcinoma; EC, endometrial carcinoma; GBM, glioblastoma; AML, acute myeloid leukemia.

**Table 2:** Pan-cancer analysis of PTEN deletion prognostic value.

Description	PTEN Loss	PTEN Del	OS *p* PTEN Loss	OS *p* PTEN Del	DFS *p* PTEN Loss	DFS *p* PTEN Del
Brain LGG	1%	22%	**2.45E-14**	**6.58E-27**	**2.15E-10**	**4.30E-24**
CCRCC	1%	18%	**3.16E-11**	2.68E-01	**1.85E-07**	1.44E-01
Pancreatic Adenocarcinoma	1%	18%	**4.70E-04**	8.23E-01	**2.23E-06**	5.87E-01
UCEC	4%	15%	**6.63E-03**	**1.78E-03**	**7.52E-05**	**1.13E-03**
Multiform GBM	10%	89%	**1.67E-02**	**1.67E-05**	**7.96E-05**	**4.26E-06**
Sarcoma	6%	54%	**1.04E-02**	1.28E-01	**6.73E-04**	1.25E-01
Head and Neck Squamous Cell Carcinoma	3%	27%	3.45E-01	7.66E-01	**4.73E-02**	7.55E-01
Prostate Adenocarcinoma	19%	32%	8.59E-01	8.33E-01	1.31E-01	7.68E-02
Bladder Urothelial Carcinoma	2%	43%	2.59E-01	9.70E-01	2.68E-01	8.37E-01
Breast Invasive Carcinoma	5%	30%	5.72E-01	4.17E-01	2.82E-01	5.97E-02
Diffuse Large B-cell Lymphoma	6%	15%	2.91E-01	3.95E-01	4.64E-01	6.38E-01
Liver Hepatocellular Carcinoma	4%	26%	9.83E-01	2.52E-01	4.92E-01	2.09E-01
Thyroid Carcinoma	1%	3%	7.20E-01	5.57E-01	5.02E-01	3.48E-01
Testicular Germ Cell Cancer	1%	46%	8.85E-01	3.19E-01	5.33E-01	2.01E-01
Colorectal Adenocarcinoma	3%	26%	1.93E-01	1.52E-01	5.33E-01	1.02E-01
Lung Squamous Cell Carcinoma	10%	54%	7.25E-02	1.79E-01	5.70E-01	2.35E-01
Skin Cutaneous Melanoma	7%	63%	2.11E-01	2.71E-01	6.04E-01	5.46E-02
Papillary Thyroid Carcinoma	1%	2%	6.85E-01	6.57E-01	6.08E-01	5.13E-01
Kidney Renal Papillary Cell Carcinoma	0%	7%	7.59E-01	**9.27E-03**	6.75E-01	**1.28E-02**
Stomach Adenocarcinoma	5%	27%	**1.96E-02**	**4.46E-02**	6.75E-01	5.17E-02
Cervical Squamous Cell Carcinoma and Endocervical Adenocarcinoma	5%	30%	6.45E-01	2.33E-01	6.92E-01	2.05E-01
Esophageal Carcinoma	4%	37%	5.00E-01	9.30E-01	7.20E-01	1.88E-01
Kidney Chromophobe	2%	74%	1.00E+00	1.70E-01	7.39E-01	4.74E-01
Lung Adenocarcinoma	1%	28%	3.02E-01	3.32E-01	7.59E-01	4.51E-01
Ovarian Serous Cystadenocarcinoma	4%	39%	4.74E-01	7.10E-02	9.93E-01	4.56E-01
AML	1%	2%	**2.83E-07**	**3.72E-10**	NA	NA
Adrenocortical Carcinoma	0%	11%	NA	2.33E-01	NA	3.50E-01
Cholangiocarcinoma	0%	17%	NA	8.36E-01	NA	9.60E-01
Esophagus-Stomach Cancers	7%	40%	9.99E-01	5.45E-01	NA	NA
Thymoma	0%	3%	NA	6.27E-01	NA	6.04E-01
Uveal Melanoma	0%	1%	NA	**6.86E-05**	NA	NA

PTEN Del, PTEN Deletion; OS, overall survival; *p*, *p*-value; DFS, disease-free survival; LGG, lower grade glioma; CCRCC, clear cell renal cell carcinoma; UCEC, uterine corpus endometrial carcinoma; AML, acute myeloid leukemia. Significant terms highlighted in bold. The survival tests were calculated via the survfit function of the “survival” R package. Patients were partitioned in each dataset accordingly to Copy Number Variation (CNV) data on the PTEN gene. Endpoint for OS was deceased/living and disease-free/recurred for DFS. PTEN Loss: genomic identification of significant targets in cancer (GISTIC) CNV score < −1. PTEN-Del: GISTIC CNV score < 0.

**Table 3:** Representative survival values for PTEN loss/del partitioning in TCGA data.

Description of Patients		OS Median			DFS Median		
Tumor Type (TCGA, Provisional)	*N*	Control	PTEN Loss	PTEN Del	Control	PTEN Loss	PTEN Del
AML	191	15.97	0.495	0	NA	NA	NA
Adrenocortical Carcinoma	90	15.97	0.495	60.84	68.96	NA	31.31
Brain LGG	513	105.12	23.88	24.9	72.17	14.13	15.97
Multiform GMB	577	17.77	12.42	13.96	14.42	6.21	7.13
Head and Neck Squamous Cell Carcinoma	522	54.89	NA	56.9	71.22	NA	61.07
CCRCC	528	90.8	3.61	84.23	123.72	2.04	88.21
Kidney Renal Papillary Cell Carcinoma	288	NA	NA	NA	106.04	NA	91.59
Lung Squamous Cell Carcinoma	501	48.78	88.04	56.27	57.72	146.88	62.81
Pancreatic Adenocarcinoma	184	20.17	15.11	19.94	17.28	3.22	14.45
Sarcoma	257	76.35	53.45	63.76	42.81	11.79	32.03
Skin Cutaneous Melanoma	367	74.67	268.53	107.06	44.65	66.03	56.8
Stomach Adenocarcinoma	441	55.39	18.33	21.98	55.06	14.09	31.6
UCEC	539	112.45	77.27	102.23	NA	62.98	69.42
Uveal Melanoma	80	112.45	NA	19.91	NA	26.77	NA

TCGA, The Cancer Genome Atlas; NA, not available; PTEN Del, PTEN deletion; OS, overall survival; DFS, disease-free survival; LGG, lower grade glioma; CCRCC, clear cell renal cell carcinoma; UCEC, uterine corpus endometrial carcinoma; AML, acute myeloid leukemia.

## Abbreviations

The following abbreviations are used in this manuscript:

AML	Acute myeloid leukemia
CCRCC	Clear cell renal cell carcinoma
CENP-C	Centromeric protein C
CIMP	CpG island methylator phenotype
CRC	Colorectal cancer
DFS	Disease-free survival
EC	Endometrial carcinoma
EEC	Endometrioid endometrial carcinoma
EGFR	Epidermal growth factor receptor
FAK	Focal adhesion kinase
FISH	Fluorescence in situ hybridation
GISTIC	Genomic identification of significant targets in cancer
GBM	Glioblastoma
IHC	Immunohistochemistry
IL	Interleukin
KIRC	Kidney renal papillary carcinoma
KRAS	Kirsten rat sarcoma
LGG	Lower grade gliomas
MAN2C1	α-mannosidase 2C1
MAPK	Mitogen-activated protein kinase
mCRC	Metastatic colorectal cancer
MEK	Mitogen-activated protein kinase kinase
miRNA	Micro RNA
MMP	Matrix metalloproteinase
MSI	Microsatellite instability
mTOR	Mammalian target of rapamycin
OS	Overall survival
PBD	PIP2-binding domain
PD-1	Programmed death-1
PD-L1	Programmed death ligand-1
PDAC	Pancreatic ductal adenocarcinoma
PI3K	PhosphatidylInositol 3-kinase
PTEN	Phosphatase and tensin homolog deleted on chromosome 10
PICT-1	Protein interacting with carboxyl terminus 1
PIP2	PhosphatIdylinositol-4,5-bisphosphate
PIP3	Phosphatidylinositol-3,4,5-trisphosphate
RCC	Renal cell carcinoma
TCGA	The cancer genome atlas
TMA	Tissue microarray
TME	Tumor microenvironment
TNM	Tumor, node, metastasis
UCEC	Uterine corpus endometrial carcinoma
